# Therapeutic drug monitoring-guided management of venlafaxine overdose in an adolescent: a case report

**DOI:** 10.1093/jat/bkag080

**Published:** 2026-09-12

**Authors:** Michaela Krivošová, Nela Žideková, Zuzana Kubisová, Tomáš Bělohlávek, Veronika Kucianová, Slavomír Nosál’, Martin Kertys

**Affiliations:** Biomedical Centre Martin, Jessenius Faculty of Medicine in Martin, Comenius University Bratislava, Malá Hora 11161/4D, Martin, 03601, Slovakia; Biomedical Centre Martin, Jessenius Faculty of Medicine in Martin, Comenius University Bratislava, Malá Hora 11161/4D, Martin, 03601, Slovakia; Clinic of Pediatric Anesthesiology and Intensive Medicine, Jessenius Faculty of Medicine in Martin, Comenius University Bratislava, Martin University Hospital, Kollárova 2, Martin, 03659, Slovakia; Clinic of Pediatric Anesthesiology and Intensive Medicine, Jessenius Faculty of Medicine in Martin, Comenius University Bratislava, Martin University Hospital, Kollárova 2, Martin, 03659, Slovakia; Clinic of Pediatric Anesthesiology and Intensive Medicine, Jessenius Faculty of Medicine in Martin, Comenius University Bratislava, Martin University Hospital, Kollárova 2, Martin, 03659, Slovakia; Clinic of Pediatric Anesthesiology and Intensive Medicine, Jessenius Faculty of Medicine in Martin, Comenius University Bratislava, Martin University Hospital, Kollárova 2, Martin, 03659, Slovakia; Department of Pharmacology, Jessenius Faculty of Medicine in Martin, Comenius University Bratislava, Malá Hora 4C, Martin, 03601, Slovakia

## Abstract

This report describes an adolescent with severe venlafaxine intoxication in whom serial therapeutic drug monitoring (TDM) was performed, enabling detailed toxicokinetic characterization throughout the clinical course. The initial plasma concentration of the active venlafaxine moiety was 7800 ng/mL, nearly 20 times the upper therapeutic reference limit. On admission, the patient presented with tachycardia, bilateral mydriasis, and generalized tonic-clonic seizures, which are typical clinical manifestations of severe venlafaxine intoxication. Due to progressive impairment of consciousness and increasing oxygen requirements, the patient underwent orotracheal intubation and remained on mechanical ventilation for nearly 120 hours. Serial measurements of venlafaxine and its active metabolite, O-desmethylvenlafaxine (ODV), provided valuable information on the toxicokinetic progression and supported clinical decision-making regarding the timing of extubation. Despite the extremely elevated venlafaxine concentration, cardiotoxicity was limited to transient QTc prolongation without ventricular arrhythmias and moderate elevations of high-sensitivity troponin I (hs-troponin I) and N-terminal pro B-type natriuretic peptide (NT-proBNP). Marked elevations of creatine kinase and myoglobin concentrations were most likely attributable to rhabdomyolysis following general tonic-clonic seizures. TDM demonstrated a progressive increase in the ODV/venlafaxine metabolite-to-parent ratio from 0.30 to 4.50, reflecting the transition from the acute intoxication phase to the elimination phase. The apparent elimination half-life was prolonged to 14.4 hours for venlafaxine and 17.7 hours for ODV. Genotyping revealed the *COMT rs4680* Met/Met genotype, which may theoretically influence catecholamine metabolism; however, its contribution to the clinical presentation remains uncertain.

## Introduction

Adolescence is a critical developmental period marked by profound emotional, psychological, and social changes that may increase susceptibility to mental disorders and self-harm behaviors, including intentional drug overdoses. The onset of many psychiatric conditions frequently occurs during this stage of life, highlighting adolescence as a particularly vulnerable period for mental health disorders. Both genetic predisposition and environmental factors contribute to the emergence of mental illness, with a family history of psychiatric illness representing an important marker of both inherited susceptibility and shared environmental influences [1]. According to the World Health Organization, suicide is the third leading cause of death among individuals aged 15–29 years [2]. Therapeutic drug monitoring (TDM) is an essential tool for optimizing drug therapy, improving treatment outcomes, and minimizing adverse effects [3]. In acute intoxications, TDM can confirm drug exposure, assess severity of intoxication and organ damage, monitor drug elimination, and help guide the management of further interventions. TDM is well established for medications with a narrow therapeutic index and variable pharmacokinetics; however, it is not routinely available for all drugs and is not universally implemented in emergency toxicology practice [3, 4].

Venlafaxine is a serotonin and noradrenaline reuptake inhibitor (SNRI) approved for the treatment of major depressive disorder (MDD), generalized anxiety disorder, panic disorder, and social phobia in adults [5]. Venlafaxine is among the antidepressants for which TDM is recommended, although the evidence supporting its clinical utility is less robust than for tricyclic antidepressants and citalopram [3, 6, 7]. The therapeutic reference range for the active venlafaxine moiety (the combined concentration of venlafaxine and O-desmethylvenlafaxine [ODV]) is 100–400 ng/mL, with a laboratory alert concentration above 800 ng/mL. Due to the considerable interindividual pharmacokinetic variability and the prolonged drug elimination following overdose, serial TDM measurements can provide valuable information for patient management [9]. This study presents a case of severe venlafaxine overdose in an adolescent, in whom serial TDM measurements were used to guide clinical management and monitor drug clearance.

## Case history

A 17-year-old female adolescent presented suddenly after becoming unresponsive following a suspected venlafaxine overdose. On the day of admission, she reportedly did not feel well; was anxious, somnolent, and reluctant to attend school. At noon, she suddenly stood up, became stiff, and collapsed. Thereafter, she remained unresponsive, with eyes open spontaneously and visible bilateral mydriasis. She sustained a laceration of her eyebrow during the fall and was transported to the Intensive Care Unit for evaluation of head trauma, as drug overdose was not initially suspected. Vital functions remained stable during transport. On admission, she was spontaneously breathing but unresponsive to painful stimuli (Glasgow Coma Scale, GCS = 7; E4V1M2), with persistent bilateral mydriasis and markedly slowed pupillary reflexes. Generalized tonic-clonic seizures subsequently developed, with more pronounced motor activity in the upper limbs. Initial clinical assessment revealed tachycardia (129 beats/min), while blood pressure, respiratory rate, oxygen saturation, and body temperature remained within normal limits. Arterial blood gas analysis and routine laboratory examinations are presented in Tables 1 and 2 and in supplementary Tables S2 and S3. Routine toxicology screening of urine samples was negative. Because of progressive deterioration in consciousness and increasing oxygen requirements, the patient underwent orotracheal intubation and was placed on mechanical ventilation with continuous analgosedation. Emergency CT of the brain and cervical spine showed no pathological lesions.

**Table 1:** Basic laboratory parameters over the course of hospitalization.

	Reference range	Day 0	Day 0	Day 1	Day 1	Day 2	Day 3	Day 4	Day 5	Day 6	Day 7	Day 8	Day 9
		14:36	20:53	5:42	11:44	5:22	5:43	5:26	5:41	5:23	5:26	5:39	5:29
Glucose	4.1–5.9 mmol/L	4.7	4.1	4.5	**7.0**	**7.9**	**7.9**	**7.3**	**6.8**	**9.1**	**8.8**	**8.7**	**8.0**
Lactate	0.6–2.4 mmol/L	**2.9**	0.7	0.8	0.9	0.7	0.6	**0.5**	1.0	1.3	-	1.1	-
Urea	2.8–7.2 mmol/L	2.8	4.5	5.4	–	**9.3**	**8.4**	6.3	6.4	6.9	**8.9**	**7.7**	**7.4**
Creatinine	48–84 μmol/L	70	**85**	**157**	**169**	**132**	83	57	50	**37**	**33**	**33**	**38**
Uric acid	155–357 μmol/L	**688**	–	–	–	251	**116**	**49**	**46**	**92**	–	**95**	**101**
AST	0.1–0.6 μkat/L	0.42	–	**0.86**	–	**0.86**	**1.16**	**2.45**	**3.17**	**5.04**	**3.06**	**2.23**	**1.53**
ALT	0.1–0.6 μkat/L	0.16	–	0.21	–	0.31	0.40	**0.64**	**1.02**	**2.15**	**2.39**	**3.00**	**3.65**
GGT	0.07–0.40 μkat/L	0.18	–	–	–	0.27	0.39	**0.76**	**2.43**	**5.91**	**5.15**	**5.11**	**5.39**
CRP	0.0–5.0 mg/L	0.2	–	**17.5**	–	**96.2**	**88.5**	**76.9**	**66.0**	**46.3**	**18.7**	**8.8**	4.1
IL-6	0.0–6.4 ng/L	**22.7**	–	**140.9**	–	**65.4**	**67.7**	**54.5**	**52.1**	0.85	–	0.49	–
eGF CKD-EPI	90–500 mL/min/1.73m^2^	110	**87**	**41**	**38**	**51**	**89**	131	136	151	156	156	149

ALT, alanine aminotransferase; AST, aspartate aminotransferase; CRP, C-reactive protein; GGT, gamma-glutamyl transferase; IL-6, interleukin 6.

Values outside the reference range are shown in bold.

**Table 2:** Cardiac and skeletal muscle enzyme levels over the course of hospitalization.

	Reference range	Day 0	Day 0	Day 1	Day 2	Day 3	Day 3	Day 4	Day 5	Day 6	Day 7	Day 8
		14:36	20:53	5:42	5:22	5:43	8:36	5:26	5:41	5:23	5:26	5:39
Creatine kinase	0.0–2.42 μkat/L	–	**54.0**	**58.4**	–	**108.5**	**143.2**	**251.5**	–	**366.7**	**109.6**	**26.7**
Creatine kinase-MB	0.0–0.4 μkat/L	**0.43**	**0.62**	**0.61**	**0.58**	–	–	–	**2.41**	**2.89**	**1.16**	**0.60**
Myoglobin	0.0–70.0 μg/L	**652.4**	**1177.3**	**628.5**	**282.7**	**2276.3**	**2813.3**	**1125.4**	**715.9**	**433.2**	**158.2**	53.7
High-sensitivity Troponin I	0.0–11.6 ng/L	9.28	–	**59.91**	**29.76**	–	**106.35**	**34.84**	**16.86**	**16.20**	–	4.18
NT-proBNP	0.0–125.0 ng/L	**354.0**	–	**287.1**	**259.2**	–	**1014.0**	**269.0**	62.4	–	–	60.8

Values outside the reference range are shown in bold.

An interview with the patient’s parents revealed that she had recently exhibited increased anxiety symptoms, although she had never been evaluated by a psychiatrist. The parents also reported that she had sent a photograph of empty medication blister packs to her friend. According to the mother, the blister packs likely contained venlafaxine medication, which was prescribed for her own treatment. Although the exact amount ingested could not be verified, the photograph suggested ingestion of up to 40 tablets (approximately 3 g of venlafaxine). The patient’s altered level of consciousness, respiratory impairment, tachycardia, mydriasis, and generalized tonic-clonic seizures, were consistent with severe venlafaxine intoxication. Based on the patient’s body weight of 43 kg, the estimated venlafaxine dose was approximately 69 mg/kg, markedly exceeding the reported toxic dose of 7 mg/kg. Considering the pharmacological properties of venlafaxine and the estimated time since the suspected ingestion, only supportive and symptomatic treatment was recommended, while extracorporeal elimination was not indicated. Following identification of venlafaxine as the probable intoxicating agent, serial TDM measurements of venlafaxine and ODV were performed to monitor the course of intoxication and drug elimination. Seven blood samples were collected over a 9-day period (Table S1, Supplementary Data).

**Figure 1:**
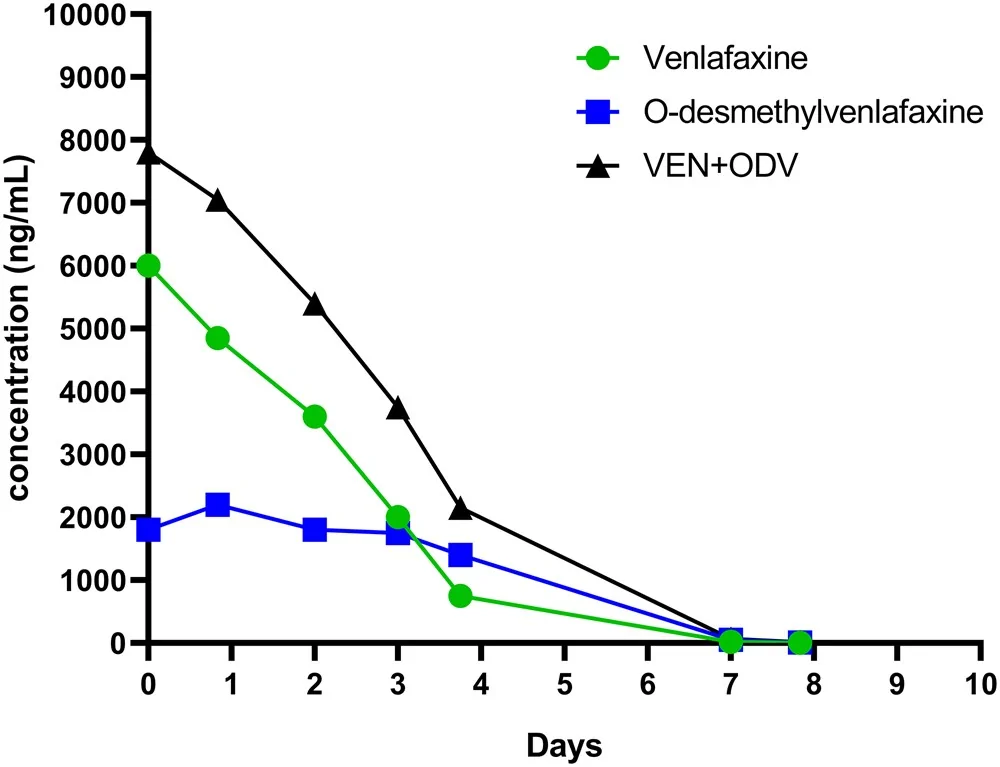
Venlafaxine and O-desmethylvenlafaxine plasma concentration changes over time. VEN+ODV, venlafaxine + O-desmethylvenlafaxine.

The initial plasma concentration of the active venlafaxine moiety was 7800 ng/mL, approximately 20-fold higher than the upper limit of the therapeutic reference range [3]. Serial TDM measurements supported clinical decision-making, particularly regarding the timing of sedation withdrawal and extubation. Changes in venlafaxine and ODV concentration over the subsequent 9 days are shown in Fig. 1. The patient remained sedated under continuous intravenous infusion of sufentanil and midazolam for 119 hours, during which gradual clinical stabilization under intensive care monitoring was achieved. Antiepileptic therapy with levetiracetam 1000 mg twice daily was continued due to ongoing seizure activity. Due to the development of severe myoglobinuria in the acute phase of intoxication (Table 2), forced diuresis using i.v. administered furosemide together with urine alkalinization using sodium bicarbonate was done as a prevention of kidney failure. On the third day of hospitalization, cardiology consultation confirmed preserved biventricular systolic function without arrhythmias, and the previously corrected prolonged QT interval (QTc; 451–485 ms) had normalized. Levetiracetam therapy was subsequently tapered and discontinued. On the fourth day, spontaneous diuresis developed, progressing to polyuric tendencies and furosemide administration was stopped. Clinical and laboratory parameters indicated gradual stabilization. On the fifth day, the active venlafaxine moiety concentration decreased to approximately 750 ng/mL. Mild elevations in hepatic transaminases (AST, ALT), gamma-glutamyl transferase (GGT), and bilirubin were observed, while myoglobin and cardiac biomarker concentrations declined in parallel with the patient’s clinical improvement. Sedative support was gradually reduced, allowing progressive neurological awakening. On the sixth day, the patient was successfully extubated after the restoration of adequate spontaneous respiratory drive and protective airway reflexes, requiring only transient supplemental oxygen thereafter. On the seventh and eighth days, the active venlafaxine moiety concentrations decreased further to 67 and 11 ng/mL, respectively, although mild elevations in hepatic enzymes (AST, ALT, GGT) persisted. On the ninth day, the patient was transferred to the Department of Psychiatry while fully conscious (GCS 15).

Genotyping demonstrated the AA (Met/Met) genotype of the *COMT rs4680* polymorphism (Val158Met), which is associated with reduced COMT enzymatic activity, potentially increasing catecholamine availability [10].

## Materials and methods

**Figure 2:**
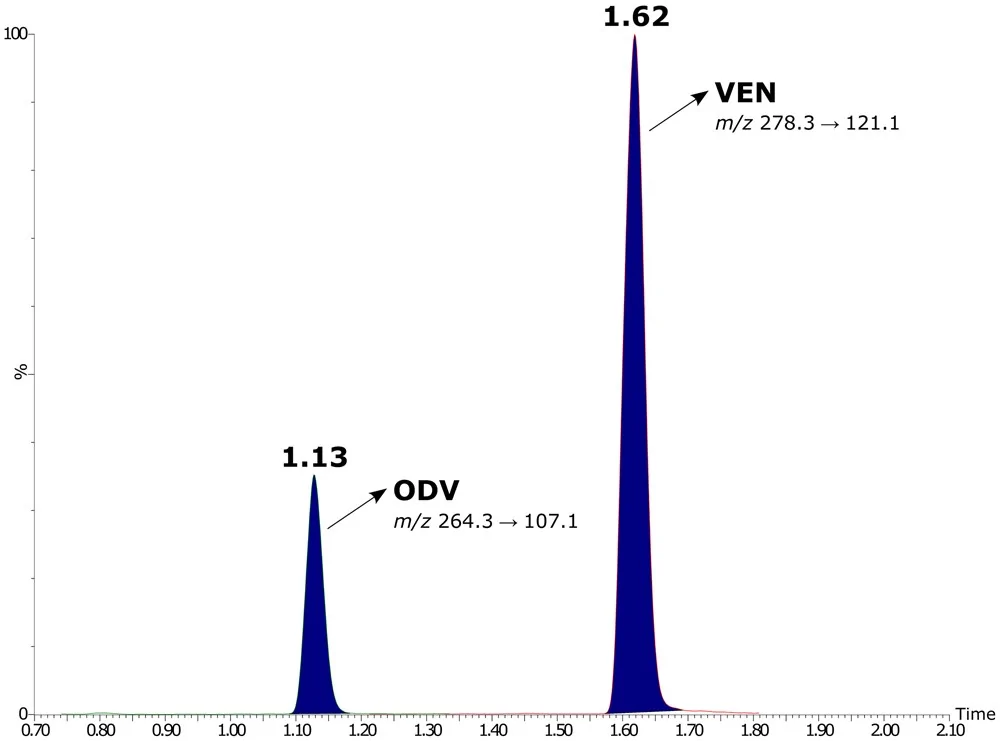
Representative chromatogram of plasma sample obtained on the day of admission. ODV, O-desmethylvenlafaxine; VEN, venlafaxine.

Laboratory parameters were assessed using routine biochemical analyses available at the hospital, while TDM measurements were performed at the Department of Pharmacology, Jessenius Faculty of Medicine in Martin, Comenius University Bratislava, using a previously published LC-MS/MS method [11]. Briefly, venlafaxine and ODV were analyzed using the ACQUITY UPLC system coupled to the XEVO TQ-S triple quadrupole mass spectrometer (Waters, Czech Republic). After a one-step extraction procedure using the OSTRO plate (Waters, Czech Republic), analytes were separated by gradient elution on an ACQUITY UPLC BEH C18 (50 × 2.1 mm, 1.7 μm; Waters) column with a runtime of 4.2 min (Fig. 2). The detection was done on a triple quadrupole tandem mass spectrometer by multiple reaction monitoring mode with transitions at *m/z* 278.3 → 121.1 for venlafaxine and *m/z* 264.3 → 107.1 for ODV using a positive electrospray ionization interface. Prior to analysis, plasma samples were appropriately diluted to ensure concentrations within the assay’s detection range.

Routine urine toxicology screening was done using the DrugLab test (DiproMed GmbH, Austria), which operates on an immunodiagnostic principle. The urine samples were tested for the detection of methadone, tetrahydrocannabinol, methamphetamine, ketamine, cocaine, benzodiazepines, barbiturates, morphine, heroin, and 3,4-methylenedioxymethamphetamine.

The QTc (corrected QT interval) was calculated using a heart rate–corrected formula to minimize the influence of heart rate on QT duration and allow comparison between measurements.

The *COMT rs4680* genotype was analyzed using a commercial Taqman Genotyping Assay (Thermo Fisher Scientific, United States) according to the manufacturer’s instructions.

## Discussion

This case report demonstrates the clinical utility of TDM in severe venlafaxine intoxication and highlights the complexity of its systemic manifestations. Serial TDM measurements not only confirmed the diagnosis but also enabled the assessment of concentration–time changes, facilitated estimation of elimination characteristics, and supported interpretation of the evolving clinical and laboratory manifestations during hospitalization.

In the setting of acute overdose, TDM can confirm drug exposure in the context of pharmacokinetic variability, quantitate drug concentrations, characterize toxicokinetic behavior, estimate elimination parameters, and help assess the relationship between drug concentrations and clinical manifestations. In the case of venlafaxine, monitoring of the active moiety (the combined concentration of venlafaxine and ODV) provides a more comprehensive assessment of overall pharmacological and toxicological exposure than evaluation of the parent compound alone [3]. Repeated TDM measurements in venlafaxine intoxication have previously been described in several adult case reports [8, 9, 12]. However, published reports of venlafaxine intoxication in children and adolescents [13, 14] have not included TDM-guided management or serial toxicokinetic monitoring. Venlafaxine is not approved for use in children or adolescents as clinical studies have demonstrated limited tolerability and an increased risk of treatment-emergent or worsening suicidality in this population [15]. Nevertheless, it could be useful in a variety of psychiatric disorders in this population, while greater treatment benefits have been reported in older adolescents [16, 17]. In venlafaxine intoxication, the most common symptoms reported include tachycardia, somnolence up to coma, mydriasis, seizures, and vomiting [13]. Venlafaxine overdose is associated with cardiotoxicity, including QT prolongation, ventricular tachycardia, and mild hypertension, resulting from excessive noradrenergic stimulation [18]. Paradoxically, severe intoxications may present with hypotension, transient myocardial dysfunction, cardiogenic shock, and the need for vasopressor support [19]. Regarding cardiotoxicity, the patient exhibited only mild-to-moderate transient QTc prolongation on ECG monitoring, with a maximum value of 485 ms on the evening of admission, despite an extremely elevated active venlafaxine moiety plasma level (7800 ng/mL at admission). The highest heart frequency (129 beats/min) was observed during the first 24 hours, and no ventricular arrhythmias were documented throughout hospitalization. This supports previous studies showing that venlafaxine overdose generally causes modest electrocardiographic abnormalities and a low incidence of malignant ventricular arrhythmias [20]. Interestingly, despite the pronounced noradrenergic activity expected in venlafaxine overdose, initial hypotension required low-dose noradrenaline support, which highlights the complex and sometimes paradoxical cardiovascular manifestations of intoxication. On the other hand, marked elevations of skeletal muscle and cardiac biomarkers were observed during the clinical course. The marked increase in creatine kinase and myoglobin levels was most likely attributable to rhabdomyolysis following generalized tonic–clonic seizures, as observed in another retrospective study [21]. Although hs-troponin I and NT-proBNP concentrations were also elevated, their increases were considerably less pronounced, suggesting a lesser degree of myocardial involvement compared with skeletal muscle injury. Nevertheless, transient QTc prolongation, elevated cardiac biomarkers, and electrocardiographic abnormalities indicate that some cardiac toxicity was present. Therefore, the elevations in cardiac biomarkers most likely reflected transient myocardial stress or mild, reversible myocardial injury rather than clinically significant cardiomyopathy. The observed cardiac manifestations were likely multifactorial, involving direct venlafaxine cardiotoxicity, catecholamine-mediated myocardial stress associated with generalized tonic–clonic seizures, and transient hypoxia during the acute intoxication phase as key contributors. Moreover, despite quite pronounced rhabdomyolysis with a peak creatine kinase concentration of 366.7 μkat/L (22002 U/L), only transient acute kidney injury was observed (maximum level of creatinine was 169 μmol/L). Renal function normalized rapidly following supportive treatment and remained preserved despite continued increases in creatine kinase concentrations.

Severe lactic acidosis was described as another less common adverse effect of venlafaxine overdose [13, 22]. In this patient, initial laboratory findings showed mild hyperlactatemia with mild metabolic acidosis, both of which later normalized. However, her estimated ingested dose was lower than those in previously published case reports. Interindividual variability in the cytochrome P450 enzymes, particularly CYP2D6, which is primarily responsible for the metabolism of venlafaxine to its major active metabolite, ODV, may also have contributed to the clinical presentation. Several studies evaluated CYP2D6 genotype in the context of venlafaxine intoxication and suggested that, together with other parameters (dose, concomitant drugs administration, comorbidities), it may contribute to the severity of intoxication [9, 23]. Younger age may also influence the rate of its metabolism [13, 24].

When considering pharmacokinetic characteristics, the reference metabolite-to-parent drug ratio (MPR) for ODV/venlafaxine under steady-state conditions ranges from 2.7 to 7.7 [3]. MPR may serve as an indirect marker of CYP2D6 activity, with low ratios typically indicating reduced conversion of venlafaxine to ODV. However, the interpretation of MPR during acute overdose is limited as the ratio is influenced by ongoing absorption, metabolite formation, and possible saturation of metabolic pathways. In the present case, MPR increased progressively from 0.30 on admission to 4.50 at the end of hospitalization. Initially, the toxicokinetic profile was characterized by a marked predominance of the parent compound, reflecting the massive venlafaxine exposure. As venlafaxine concentrations declined, the relative contribution of ODV increased, resulting in a gradual shift toward metabolite predominance. This dynamic change in MPR illustrates the transition from the acute intoxication phase to the elimination phase and provides additional insight into the metabolic progression of venlafaxine overdose. The progressive increase in the ODV/venlafaxine ratio suggests that metabolic conversion of venlafaxine to ODV is preserved. Nevertheless, due to the massive overdose and the non-steady-state conditions, no conclusions regarding CYP2D6 metabolizer status can be drawn from these data alone.

At therapeutic doses, venlafaxine and ODV have an elimination half-life of approximately 5 and 11 hours, respectively [8]. In this patient, the apparent elimination half-life calculated from the terminal phase (samples collected on Days 3, 4, and 7) was approximately 14.4 hours for venlafaxine and 17.7 hours for ODV, indicating a substantial prolongation compared with therapeutic conditions (supplementary Table S4). Although concomitant medications administered during intensive care may theoretically influence venlafaxine pharmacokinetics, none is likely to fully account for the observed elimination profile.

Other symptoms included mild stress hyperglycemia, transient hypokalemia, and mild elevations of liver enzymes. Hyperglycemia observed during hospitalization most likely reflected the combined effects of acute intoxication, generalized tonic–clonic seizures, systemic inflammatory activation, and critical illness, while concomitant dexamethasone therapy for post-extubation dysphonia and dysphagia could further contribute to elevated glucose levels. The increase in AST closely paralleled creatine kinase concentrations and was therefore likely attributable, at least in part, to skeletal muscle injury associated with rhabdomyolysis. In contrast, the delayed elevation of ALT and GGT may suggest a minor contribution of hepatic stress or transient hepatocellular injury related to severe intoxication and critical illness. Importantly, no evidence of clinically significant liver dysfunction was observed, and liver enzyme abnormalities gradually resolved during recovery.

Regarding pharmacogenetic analysis, the patient was found to carry the *COMT* rs4680 AA (Met/Met) genotype, which is associated with reduced catechol-O-methyltransferase activity and thus slower catecholamine degradation [10]. Given the pronounced noradrenergic effects of venlafaxine at toxic concentrations, this genotype could theoretically contribute to enhanced sympathetic activation during intoxication. Nevertheless, despite extremely high venlafaxine concentrations and evidence of transient myocardial stress, no malignant arrhythmias or clinically significant cardiac dysfunction were observed. However, the clinical relevance of the COMT genetic variation in venlafaxine-induced cardiotoxicity remains unclear and warrants further investigation.

## Conclusion

This case highlights the clinical value of serial TDM in the management of severe venlafaxine intoxication in an adolescent. Repeated measurements of venlafaxine and ODV concentrations supported further clinical management of this patient. Despite extremely high venlafaxine concentrations, only transient QTc prolongation and mild reversible cardiac involvement were observed, without malignant ventricular arrhythmias or persistent myocardial dysfunction. In contrast, generalized tonic–clonic seizures were followed by severe rhabdomyolysis and transient acute kidney injury, representing the most prominent toxic manifestations. This case demonstrates that severe venlafaxine overdose may result in substantial multiorgan toxicity even in the absence of life-threatening arrhythmias and supports the use of serial TDM as a valuable adjunct for diagnosis, toxicokinetic assessment, and clinical interpretation of intoxication severity.

## Supplementary Material

bkag080_Supplementary_Data

## Data Availability

All data are incorporated into the article and its online supplementary material.
