# Supplementary material for: Therapeutic drug monitoring-guided management of venlafaxine overdose in an adolescent: a case report
Source: J Anal Toxicol. 2026 Sep 12;50(9):bkag080. doi: 10.1093/jat/bkag080 (PMC13616046; doi:10.1093/jat/bkag080)
Supplement: Supplementary file 1 [file bkag080_supplementary_data.zip › jat-26-4932-File005.docx]

**Therapeutic Drug Monitoring-Guided Management of Venlafaxine Overdose in an Adolescent: a case report**

**Supplementary data**

**Table S1. Serial therapeutic drug monitoring results of venlafaxine and O-desmethylvenlafaxine**

| Time | Venlafaxine (ng/mL) | ODV (ng/mL) | Venlafaxine + ODV (ng/mL) | MPR |
| --- | --- | --- | --- | --- |
| Day 0 | 6000 | 1800 | 7800 | 0.30 |
| Day 1 (+ 20 hrs) | 4850 | 2200 | 7050 | 0.45 |
| Day 2 (+ 48 hrs) | 3600 | 1800 | 5400 | 0.50 |
| Day 3 (+ 72 hrs) | 2000 | 1750 | 3750 | 0.88 |
| Day 4 (+ 90 hrs) | 750 | 1400 | 2150 | 1.87 |
| Day 7 (+ 168 hrs) | 19 | 48 | 67 | 2.53 |
| Day 8 (+ 188 hrs) | 2 | 9 | 11 | 4.50 |

MPR – metabolite-to-parent drug ratio, ODV – O-desmethylvenlafaxine

**Table S2.** **Hemogram parameters over the course of hospitalisation**

|  | Reference range | Day 1 | Day 2 | Day 3 | Day 4 | Day 5 | Day 6 | Day 7 | Day 8 | Day 9 |
| --- | --- | --- | --- | --- | --- | --- | --- | --- | --- | --- |
|  |  | 5:13 | 5:22 | 3:54 | 5:17 | 3:15 | 5:11 | 5:13 | 5:18 | 5:01 |
| White blood cell | 3.9-10.0×10^9^/L | **13.8** | **10.9** | 9.1 | 8.1 | 6.1 | 7.3 | 9.0 | 7.7 | 7.7 |
| Red blood cell | 3.8-5.2×10^12^/L | **3.65** | **3.56** | **3.60** | **3.52** | **3.43** | **3.20** | **3.20** | **3.22** | **3.50** |
| Hemoglobin | 120-155 g/L | **106** | **107** | **102** | **101** | **102** | **95** | **91** | **95** | **99** |
| Hematocrit | 0.36-0.47 | **0.32** | **0.31** | **0.32** | **0.31** | **0.30** | **0.28** | **0.28** | **0.28** | **0.30** |
| Platelet | 140-400×10^9^/L | 243 | 222 | 185 | 188 | 200 | 210 | 218 | 271 | 290 |
| Red Cell Distribution Width | 11.6-14.5% | **14.8** | **15.1** | **15.8** | **15.1** | **14.8** | 14.2 | 14.4 | 14.4 | 14.3 |
| Neutrophil | 1.4-6.5×10^9^/L | **12.49** | **9.03** | **7.44** | 6.18 | 4.06 | **6.71** | **7.9** | 6.32 | 6.12 |
| Lymphocyte | 1.2-3.4×10^9^/L | **0.64** | **0.74** | **0.82** | **0.93** | **1.18** | **0.36** | **0.58** | **0.71** | **1.08** |
| Monocyte | 0.08-0.7×10^9^/L | **0.51** | **0.80** | 0.67 | **0.72** | 0.53 | **0.18** | 0.47 | 0.63 | 0.46 |

*Values outside the reference range are shown in bold.*

**Table S3.** **Blood gas parameters over the course of hospitalisation**

|  | Reference  range | Day 0 | Day 0 | Day 1 | Day 1 | Day 2 | Day 3 | Day 4 | Day 5 | Day 6 | Day 8 | Day 9 |
| --- | --- | --- | --- | --- | --- | --- | --- | --- | --- | --- | --- | --- |
|  |  | 14:36 | 20:53 | 5:42 | 11:44 | 5:22 | 5:43 | 5:26 | 5:41 | 5:23 | 5:39 | 5:29 |
| pH | 7.360-7.440 | **7.338** | **7.458** | 7.425 | **7.442** | 7.435 | 7.400 | 7.412 | 7.425 | **7.443** | 7.369 | **7.447** |
| pCO_2_ | 4.64-6.0 kPa | 5.34 | **4.22** | 4.74 | 4.91 | 4.95 | 5.31 | 5.20 | 4.90 | 4.82 | **6.39** | 4.85 |
| pO_2_ | 9.8-13.3 kPa | **15.90** | **16.19** | 12.67 | **13.85** | **19.12** | **16.73** | **19.38** | **8.94** | 13.09 | **5.32** | 12.31 |
| HCO_3_^-^ | 22-26 mmol/L | **21.0** | **21.9** | 22.8 | 24.5 | 24.3 | 24.1 | 24.3 | 23.6 | 24.2 | **27.0** | 24.5 |

*Values outside the reference range are shown in bold.*

**Table S4.** **Parameters of apparent elimination half-time calculation**

| Analyte | Selected time points (hrs) | Concentration (ng/ml) | Slope | R^2^ | Apparent t_1/2_ (hrs) |
| --- | --- | --- | --- | --- | --- |
| Venlafaxine | 72 – 168 | 2000 – 19 | -0.0481 | 0.9994 | 14.41 |
| O-desmethylvenlafaxine | 72 – 168 | 1750 – 48 | -0.0392 | 0.9852 | 17.69 |

R^2^ – coefficient of determination, t_1/2_ – elimination half-time

A semilogarithmic plasma concentration-time plot was created to identify the apparent terminal log-linear phase. Based on visual inspection of this plot, linear regression of the natural logarithm of plasma concentrations versus time was performed to estimate the apparent elimination half-life of venlafaxine and O-desmethylvenlafaxine (ODV) using the concentration measurements obtained at time points +72 hrs, +90 hrs, and +168 hrs. These time points were selected because the earlier measurements were considered to represent the distribution phase rather than the terminal elimination phase (Jeong and Jusko, 2022), whereas the final measurement (+188 hrs) was close to the lower limit of quantification and therefore was not considered sufficiently reliable for estimation of the terminal elimination rate constant. The terminal elimination rate constant (k_e_) was obtained from the slope of the linear regression, and the apparent elimination half-life was calculated as t_1/2_=ln(2)/k_e_. The selected time points, concentration ranges, regression slopes, coefficients of determination (R²), and the resulting apparent half-life estimates are presented in Supplementary Table 4. The calculation was performed in GraphPad Prism 8.0.1 (GraphPad, San Diego, CA, USA).

*Jeong YS, Jusko WJ. Determinants of Biological Half-Lives and Terminal Slopes in Physiologically Based Pharmacokinetic Systems: Assessment of Limiting Conditions. AAPS J. 2022;24(5):96. doi:10.1208/s12248-022-00739-5*
